# Correction: Bai et al. A Novel Steganography Method for Infrared Image Based on Smooth Wavelet Transform and Convolutional Neural Network. *Sensors* 2023, *23,* 5360

**DOI:** 10.3390/s26185858

**Published:** 2026-09-16

**Authors:** Yu Bai, Li Li, Jianfeng Lu, Shanqing Zhang, Ning Chu

**Affiliations:** 1 School of Computer Science and Technology, Hangzhou Dianzi University, Hangzhou 310018, China; baiyu@hdu.edu.cn (Y.B.); lili2008@hdu.edu.cn (L.L.); jflu@hdu.edu.cn (J.L.); 2 Zhe-Jiang Shangfeng Special Blower Company Ltd., Shaoxing 312352, China; chuning1983@sina.com

**References Correction**

In the original publication [1], References 7–16, 24, 27, 40, and 41 were identified as not directly supporting the corresponding in-text statements and have therefore been replaced with new references that better support the in-text claims. 

7. Zhao, J.; Chen, Y.; Feng, H.; Xu, Z.; Li, Q. Infrared Image Enhancement through Saliency Feature Analysis Based on Multi-scale Decomposition. *Infrared Phys. Technol*. **2014**, *62*, 86–93. https://doi.org/10.1016/j.infrared.2013.11.008. 

8. Qi, Y.; He, R.; Lin, H. Novel Infrared Image Enhancement Technology Based on the Frequency Compensation Approach. *Infrared Phys. Technol*. **2016**, *76*, 521–529. https://doi.org/10.1016/j.infrared.2016.03.021. 

9. Li, H.; Wu, X.J. DenseFuse: A Fusion Approach to Infrared and Visible Images. *IEEE Trans. Image Process*. **2018**, *28*, 2614–2623. https://doi.org/10.1109/TIP.2018.2887342. 

10. Li, H.; Wu, X.-J.; Kittler, J.; Wang, Z. Infrared and Visible Image Fusion Using a Deep Learning Framework. In Proceedings of the 2018 24th International Conference on Pattern Recognition (ICPR), Beijing, China, 20–24 August 2018; pp. 2705–2710. https://doi.org/10.1109/ICPR.2018.8546006. 

11. Xu, H.; Ma, J.; Jiang, J.; Guo, X.; Ling, H. U2Fusion: A Unified Unsupervised Image Fusion Network. *IEEE Trans. Pattern Anal*. *Mach. Intell.* **2022**, *44*, 502–518. https://doi.org/10.1109/TPAMI.2020.3012548. 

12. Zhong, S.; Fu, L.; Zhang, F. Infrared Image Enhancement Using Convolutional Neural Networks for Auto-Driving. *Appl. Sci*. **2023**, *13*, 12581. https://doi.org/10.3390/app132312581.

13. Tang, W.; Li, B.; Tan, S.; Barni, M.; Huang, J. CNN-Based Adversarial Embedding for Image Steganography. *IEEE Trans. Inf. Forensics Secur.* **2019**, *14*, 2074–2087. https://doi.org/10.1109/TIFS.2019.2891237. 

14. Zhu, J.; Kaplan, A.; Johnson, J.; Li, F.-F. HiDDeN: Hiding Data with Deep Networks. In Proceedings of the European Conference on Computer Vision (ECCV), Munich, Germany, 8–14 September 2018; pp. 682–697. https://doi.org/10.1007/978-3-030-01267-0_40. 

15. Wani, M.A.; Sultan, B. Deep Learning Based Image Steganography: A Review. *WIREs Data Min. Knowl. Discov*. **2023**, *13*, e1481. https://doi.org/10.1002/widm.1481. 

16. Yang, J.; Ruan, D.; Huang, J.; Kang, X.; Shi, Y. An Embedding Cost Learning Framework Using GAN. *IEEE Trans. Inf. Forensics Secur*. **2020**, *15*, 839–851. https://doi.org/10.1109/TIFS.2019.2922229. 

24. Yang, X.; Huang, F. New CNN-Based Predictor for Reversible Data Hiding. *IEEE Signal Process. Lett*. **2022**, *29*, 2627–2631. https://doi.org/10.1109/LSP.2022.3231193. 

27. Ellmauthaler, A.; Pagliari, C.L.; da Silva, E.A.B. Multiscale Image Fusion Using the Undecimated Wavelet Transform With Spectral Factorization and Nonorthogonal Filter Banks. *IEEE Trans. Image Process*. **2013**, *22*, 1005–1017. https://doi.org/10.1109/TIP.2012.2226045. 

40. Ou, B.; Li, X.; Zhao, Y.; Ni, R.; Shi, Y.Q. Pairwise Prediction-Error Expansion for Efficient Reversible Data Hiding. *IEEE Trans. Image Process*. **2013**, *22*, 5010–5021. https://doi.org/10.1109/TIP.2013.2281422. 

41. Li, X.; Zhang, W.; Gui, X.; Yang, B. Efficient Reversible Data Hiding Based on Multiple Histograms Modification. *IEEE Trans. Inf. Forensics Secur*. **2015**, *10*, 2016–2027. https://doi.org/10.1109/TIFS.2015.2444354. 

With this correction, the order of some references has been adjusted accordingly. 

**Text Correction**

1. In the 2nd Paragraph of Section 1. The content “Unlike the multi-scale transform that was applied in [5,6], Li et al. [7] decomposed infrared images with visible images based on Latent Low-Rank Representation (LatLRR). The above-mentioned algorithms used traditional methods for infrared- and visible-images fusion. After Luo et al. [8] obtained infrared images using a Fourier Transform Infrared Spectrometer, they analyzed the changes in image details to grasp the evolution of specific microstructures.” has been updated to “Unlike the multi-scale transform that was applied in [5,6], researchers have also focused on extracting subtle high-frequency details from infrared images to achieve image enhancement and feature extraction. For example, Zhao et al. [7] proposed an infrared image enhancement algorithm based on multiscale decomposition and saliency feature analysis to effectively preserve thermal radiation details. Furthermore, to address noise and low contrast issues in the frequency domain. Qi et al. [8] developed a frequency compensation method to improve the visual quality and feature representation capabilities of infrared images. The aforementioned algorithms all employ traditional multiscale and frequency-domain methods to achieve feature extraction and enhancement of infrared images.”
2. In the 3rd Paragraph of Section 1. The content “Image-feature extraction has a very important position in all of the above algorithms. Thus, Wang et al. [9] improved the accuracy of feature extraction based on the ensemble-learning algorithm. Convolution Neural Networks (CNNs) are able to extract features of images very intelligently. Compared with traditional algorithms, CNN has better results in image-feature extraction. For example, Ao et al. [10] used CNN to extract images of patient-specific organs for local treatment. Fu et al. [11] proposed the depth-estimation algorithm for extracting simple and complex textures based on CNN. Wang et al. [12] investigated a multispecies transferable algorithm to improve the prediction accuracy of the CNN model. Zhang et al. [13] proposed a new classifier applied to an electroencephalogram for the overfitting phenomenon of CNN-model features. Deng et al. [14] applied CNNs to the field of natural-language processing to solve the challenge of non-action question and answer. Liu et al. [15] found that the global perception capability of CNNs needs to be improved. Therefore, they combined a Fast Fourier Transform with CNN to improve the performance of the model. Moreover, CNN has derived more novel models in various research areas in the last decade.” has been updated to “Image feature extraction plays a crucial role in advanced image processing tasks, particularly in the fields of infrared image analysis and image steganography. Convolutional neural networks (CNN) have demonstrated exceptional capabilities in intelligently extracting deep semantic and textural features, with performance far surpassing that of traditional, manually designed algorithms. In the field of infrared and visible light image fusion, CNN-based architectures have been widely used to extract complementary features from multimodal sensors, thereby synthesizing images with prominent targets and rich details [9,10]. Furthermore, deep learning frameworks have been specifically designed to capture thermal radiation and textural features in infrared images to achieve robust image enhancement and target detection [11,12]. In the fields of image steganography and watermarking, convolutional neural networks have revolutionized the embedding and extraction of secret information by learning the complex feature space of the host image, ensuring a high degree of imperceptibility and robustness [13,14]. A series of recent comprehensive studies has also emphasized that the deep feature representations learned by CNNs are crucial for optimizing embedding capacity and visual quality in modern steganography schemes [15]. Therefore, leveraging the powerful feature extraction capabilities of CNNs, which provides a solid foundation for improving the prediction accuracy and performance of infrared image steganography.”
3. In the 4th Paragraph of Section 1. The content “Ma et al. [16] proposed the FusionGAN model, which is capable of fusing images with different resolutions. The FusionGAN generator first classified the features of infrared images and then fused them with visible images. The discriminator ensured that the fused images had more details of visible images.” has been updated to “For example, Yang et al. [16] proposed an embedding cost learning framework using a GAN to automatically learn optimal embedding probabilities and enhance steganographic security.”
4. In the 5th Paragraph of Section 1. The content “Weng et al. [24] discovered that most of the improved PVO-based algorithms are applied to predict the pixels that occupy only a small fraction of the image block. Thus, they proposed a Dynamic Improved PVO (DIPVO) algorithm based on graded local complexity to dynamically hide information.” has been updated to “To overcome the limitations of traditional manual prediction model construction, recent studies have introduced deep learning models to further reduce prediction errors. For example, Yang et al. [24] proposed a novel prediction model based on CNN, which leverages the powerful feature extraction capabilities of neural networks to capture complex spatial dependencies and optimize the distribution of prediction errors.”
5. In the 6th Paragraph of Section 1. The content “This step refers to the image-super-resolution algorithm based on signal preprocessing proposed by Huang et al. [27]. Then, we apply Smooth Wavelet Transform (SWT) to extract high- and low-frequency sub-bands of the image.” has been updated to “Then, we apply Smooth Wavelet Transform (SWT) [27] to extract high- and low-frequency sub-bands of the image.”
6. In the 3rd Paragraph of Section 3.1.4. The content “For example, in Figure 6b, since the nearest zero point was located to the right of the peak, the watermark containing histogram had more pixels to the right of 0.” has been updated to “For example, in Figure 6b, since the nearest zero point was located to the right of the peak, the watermarked histogram had more pixels to the right of 0.”
7. In the 1st Paragraph of Section 4.2. The content “To evaluate the prediction-accuracy performance of the proposed algorithm, we used the mean absolute value, variance, and MSE of the image-prediction errors for the proposed SSCNNP model, the CNNP model, and three state-of-the-art algorithms: BIP [39], MEDP [40], and GAP [41]. Table 1 shows that the proposed SSCNNP had a good prediction accuracy compared with CNNP, BIP [39], MEDP [40], and GAP [41]. For the three evaluation indicators shown in Table 1 below, the lower the values, the more accurate the prediction was.” has been updated to “To evaluate the prediction-accuracy performance of the proposed algorithm, we used the mean absolute value, variance, and MSE of the image-prediction errors for the proposed SSCNNP model, the CNNP model, and three state-of-the-art algorithms: BIP [39], PEEP [40], and MHMP [41]. Table 1 shows that the proposed SSCNNP had a good prediction accuracy compared with CNNP, BIP [39], PEEP [40], and MHMP [41]. For the three evaluation indicators shown in Table 1 below, the lower the values, the more accurate the prediction was.”
8. In the 2nd Paragraph of Section 4.2. The content “As we can see from Table 1, the absolute value of the mean prediction error of the proposed SSCNNP model was only 2.04, which was lower than that of the CNNP model (2.72), BIP (4.19), MEDP (5.24), and GAP (6.59). Moreover, the variance of the proposed SSCNNP model was only 28.64, which was lower than that of CNNP model (37.15), BIP (62.48), MEDP (103.19), and GAP (136.42). For MSE, the proposed SSCNNP model achieved 36.91, which was lower than the CNNP model (59.37), BIP (93.60), MEDP (148.43), and GAP (207.32). Table 1 indicates that the proposed SSCNNP model outperformed the others on image-prediction accuracy.” has been updated to “As we can see from Table 1, the absolute value of the mean prediction error of the proposed SSCNNP model was only 2.04, which was lower than that of the CNNP model (2.72), BIP (4.19), MEDP (5.24), and GAP (6.59). Moreover, the variance of the proposed SSCNNP model was only 28.64, which was lower than that of CNNP model (37.15), BIP (62.48), MEDP (103.19), and GAP (136.42). For MSE, the proposed SSCNNP model achieved 36.91, which was lower than the CNNP model (59.37), BIP (93.60), MEDP (148.43), and GAP (207.32). Table 1 indicates that the proposed SSCNNP model outperformed the others on image-prediction accuracy.”
9. In the 3rd Paragraph of Section 4.2. The content “Figure 9 shows the prediction errors of the proposed SSCNNP, CNNP, BIP [39], MEDP [40], and GAP [41] for the four infrared images shown in Figure 8. The higher the vertical coordinate of the model in the figure at the position where the prediction error was 0, then the better the model was. For better visualization, only the prediction errors within the range [–10, 10] are shown. From Figure 9, it was found that the prediction error histogram of the proposed SSCNNP model outperformed the other methods. In particular, the proposed SSCNNP model had more pixels with accurate predictions for the prediction error in the range [–1, 1]. The BIP, MEDP, and GAP algorithms use a single convolution-kernel feature extraction and pixel prediction.” has been updated to “Figure 9 shows the prediction errors of the proposed SSCNNP, CNNP, BIP [39], PEEP [40], and MHMP [41] for the four infrared images shown in Figure 8. The higher the vertical coordinate of the model in the figure at the position where the prediction error was 0, then the better the model was. For better visualization, only the prediction errors within the range [–10, 10] are shown. From Figure 9, it was found that the prediction error histogram of the proposed SSCNNP model outperformed the other methods. In particular, the proposed SSCNNP model had more pixels with accurate predictions for the prediction error in the range [–1, 1]. The BIP algorithms use a single convolution-kernel feature extraction and pixel prediction. And the PEEP and MHMP haven’t used the CNN for image prediction.”
10. In the 1st Paragraph of Section 4.3. The content “In this section, we introduce a performance analysis of the proposed SSCNNP compared with CNNP, LPVO [42], IPPVO [25], and BLTM [43] for information hiding with different watermark capacities on the two test datasets, as listed in Tables 2 and 3.” has been updated to “In this section, we introduce a performance analysis of the proposed SSCNNP compared with CNNP, LPVO [25], IPPVO [42], and BLTM [43] for information hiding with different watermark capacities on the two test datasets, as listed in Tables 2 and 3.”
11. In the 1st Paragraph of Section 4.3.1. The content “Table 2 shows the PSNR values of the proposed algorithm compared with CCNP [28], LPVO [42], IPPVO [25], and BLTM baiyu@hdu.edu.cn 43] when the watermarking capacity was 10,000 bits and 20,000 bits.” has been updated to “Table 2 shows the PSNR values of the proposed algorithm compared with CCNP [28], LPVO [25], IPPVO [42], and BLTM [43] when the watermarking capacity was 10,000 bits and 20,000 bits.”
12. The word “ReLu” has been updated to “ReLU” across the paper.

**Table Correction**

Due to reference replacements, Table 1’s content has been updated to:

**Image Correction**

**Figure 9:**
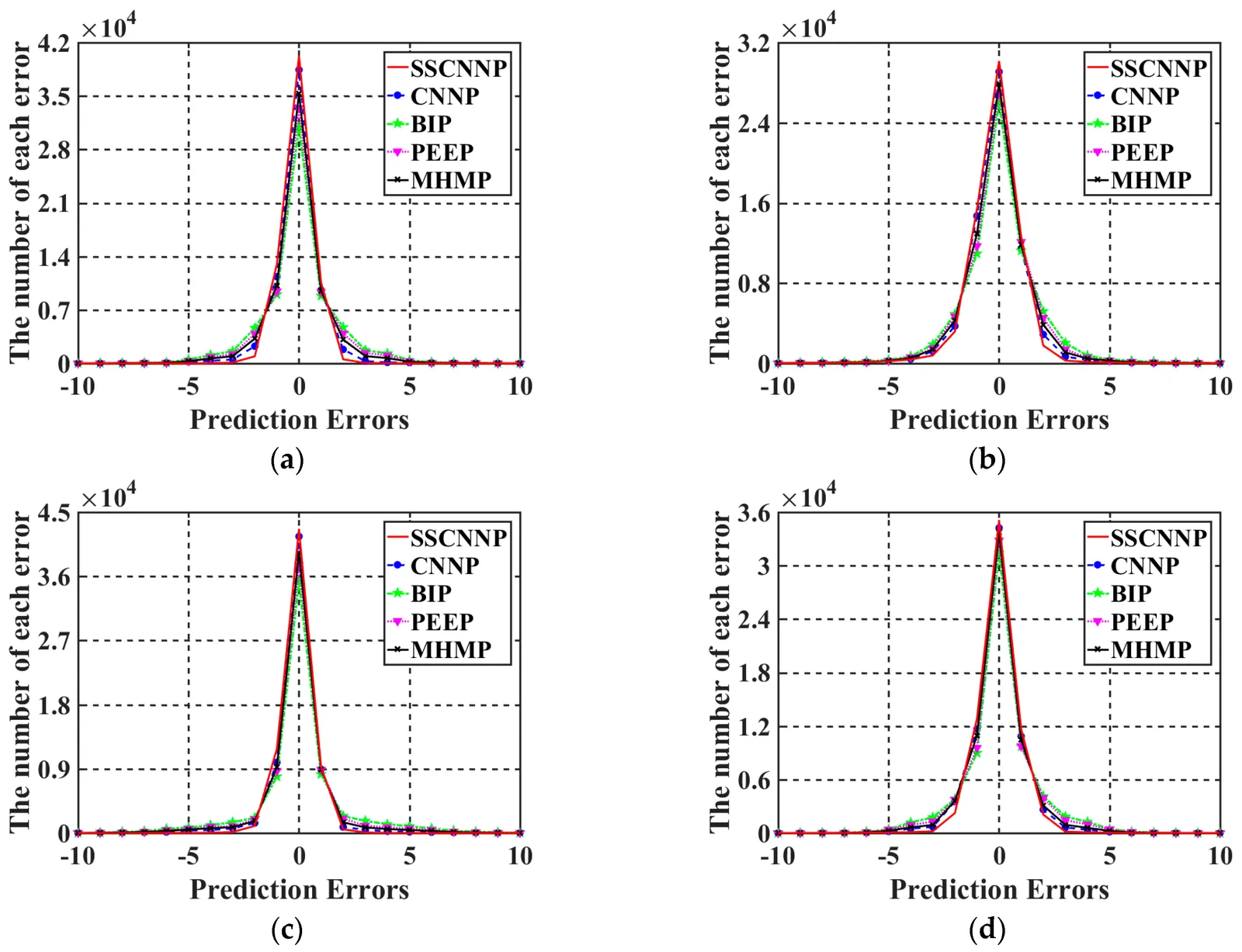
The prediction errors for the four infrared images shown in Figure 8. (**a**–**d**) correspond to the four subfigures in Figure 8, respectively.

The authors state that the scientific conclusions are unaffected. This correction was approved by the Academic Editor. The original publication has also been updated.

## Figures and Tables

**Table 1:** Mean, variance, and MSE prediction errors in the testing set. Boldface indicates best performance.

Predictor	SSCNNP	CNNP [28]	BIP [39]	PEEP [40]	MHMP [41]
Mean	2.05	2.72	4.19	3.61	3.27
Variance	28.64	37.15	62.48	59.13	51.76
MSE	36.91	59.37	93.60	78.46	64.82
